# Russian honey bee genotype identification through enhanced marker panel set

**DOI:** 10.3389/finsc.2022.998310

**Published:** 2022-11-21

**Authors:** Arian Avalos, Lelania Bilodeau

**Affiliations:** Honey Bee Breeding, Genetics, and Physiology Research Laboratory, USDA-ARS, Baton Rouge, LA, United States

**Keywords:** honey bee (*Apis mellifera L.*), genetic stock identification, Russian honey bees, population identification, machine learning, classification probability estimation

## Abstract

Russian honey bees (RHB) are a breeding population developed by USDA-ARS as an effort to provide *Varroa*-resistant honey bees to beekeepers. The selection strategy for this breeding population was the first in honey bees to incorporate genetic stock identification (GSI). The original GSI approach has been in use for over a decade, and though effective, novel technologies and analytical approaches recently developed provide an opportunity for improvement. Here we outline a novel genotyping assay that capitalizes on the markers used in the GSI as well as new loci recently identified in a whole genome pooled study of commercial honey bee stocks. Our approach utilizes a microfluidic platform and machine learning analyses to arrive at an accurate, high throughput assay. This novel approach provides an improved tool that can be readily incorporated into breeding decisions towards healthier more productive bees.

## Introduction

The Russian honey bee (RHB) breeding population was established by the USDA-ARS Honey Bee Breeding, Genetics & Physiology Laboratory (HBBGPL) after importing naturally *Varroa*-resistant RHB in 1997 (1, 2). A closed breeding system was developed, resulting in 18 distinct lines. These breeding lines were later grouped into 3 blocks used in a cross-breeding mating strategy to maintain the population. Initial breeding efforts focused on improving resistance to tracheal mites (*Acarapis woodi*) and *Varroa* mites (*Varroa destructor*), in addition to high honey production (3–8). Further refinement of the breeding population culminated in a release to the Russian Bee Breeders Association (RBBA) in 2008. As part of the release, a GSI assay was developed to discriminate the selected RHB population from other commercially available honey bee populations throughout the U.S. (9).

The GSI assay incorporated allele frequency data from 11 microsatellite and 5 single nucleotide polymorphism (SNP) loci to accurately identify RHB breeding lines of honey bees (9). Broadly, the analysis used a reference baseline sample set of known RHB honey bees and a separate sample set collected from several commercial non-Russian honey bee populations across the U.S. with the aim of maximizing diversity. The GSI assay then estimated the likelihood of membership to either group, using a similar empirical approach to that applied in salmonid fisheries population identification (10–12). When applied to honey bees, the method provided accurate and consistent assignment with only 8 worker samples per colony (9). Ultimately this method was incorporated by the RBBA to complement their multi-trait selection program, becoming the first major genetic certification assay of a honey bee population and indeed the only one for insect breeding.

Since development, the assay has been refined due to shifts in allele frequencies. The present GSI assay derives likelihoods using only 9 of the original microsatellite and 2 of the original SNPs (13). This is a major current limitation of the GSI assay, the reduced number of reliable markers coupled with high recombination rate of the honey bee genome (14) and documented introgression event in RHB (13) has added a greater degree of uncertainty. Continued loss of information from marker alleles could eventually make the GSI assay unreliable. In addition, there are also logistic limitations to using the GSI assay. Principal among these is that the assay itself is time consuming, labor-intensive, and low throughput when compared to the projected output in a microfluidic system.

Genotyping *via* a microfluidic platform has been a recent advance in marker-based analyses. Most DNA genotyping approaches (original GSI included) are conducted at a processing scale that is still defined by a human operator. At such scale the genotyping is often made more efficient by scaling up the quantity of reactions or parallelizing the steps in the method. A microfluidic system further improves on this by miniaturizing the chemistry using computer-chip inspired arrays. This approach retains all of the features of a standard DNA genotyping approach, while also allowing for a greater number of chemical reactions at one time with significantly less reagent waste (15).

In this study we outline the development and application of an expanded GSI assay. Using informative markers from the original GSI panel and novel markers obtained from recent whole-genome pooled sequencing of many of the honey bee populations in the U.S. (16) we are able to increase the degree of resolution with which we examine this important breeding population. Furthermore, by capitalizing on novel microfluidic technologies and machine learning analytical approaches to group identification, our approach can quickly and accurately provide high throughput results to arrive at reliable stock identification decisions.

## Methods

### Data sets

Using two pre-existing data sets (9, 16) we selected a panel of biallelic SNP markers that can reliably segregate the RHB population from the baseline genetic diversity across the US. In (9) researchers identified 16 markers capable of segregating the RHB population from a diverse pool of samples gathered from commercial and research populations across the US. More recently this set has been reduced to 11 markers (13) and we used these as our focal reference. Similarly, (16) examined pooled whole genome sequencing of commercial and research honey bee populations which included many of those populations previously used for the GSI development (9).

Differences in our reference datasets necessitated study-specific strategies for identifying and extracting likely candidate SNPs. As a genome-wide survey of genetic diversity, the study by Saelao and colleagues afforded a greater resolution as well as a larger number of markers to be considered in our study. We used the freely available sequencing data in (16) (BioProject # PRJNA605407) but followed an alternative method for variant calling. Specifically, our approach followed GATK best practices (17) and used a joint variant calling step in lieu of the population-specific variant calling used by Saelao and colleagues. This modification was necessary as the population analysis in (16) conservatively focused on the common variation across populations, while an identification assay would most benefit from population-specific variation. Variant calling arrived at a joint set of 3,640,438 biallelic SNPs across the honey bee genome. For each of these ~3.6 M markers we extracted the allele frequency matrix derived from SNP by sample read counts and calculated a pooled sample fixation index (F_ST_) for each marker. In our approach F_ST_ was estimated in a one-to-many comparison between the RHB population and all others in the data set. In this way we identified markers with divergent allele frequencies unique to the RHB population. All F_ST_ calculations were conducted using the *poolfstat* R package (18, 19). The final subset of SNPs with outlier F_ST_ values derived from (16) totaled 177 putative SNPs.

To extract discriminant biallelic markers from the data set in (9) we applied a linkage-based identification approach. Of the current set of eleven markers (9), nine are microsatellites which correspond to structural variants, specifically short sequence repeats (SSRs), that do not directly translate to a SNP-based assay directly. However, by identifying adjacent SNPs in direct linkage with microsatellite features, we can retain the reliability of such markers. To achieve adjacent SNP identification, we used *blastn* (20) to localize the original primer sequences to the new the honey bee genome assembly (21). The localized paired primer sets allowed us to estimate the amplicon spans across the new reference genome, and we overlapped these with known regions of low recombination (haplotype blocks) across the honey bee genome (22). A total of 13 haplotype blocks were identified to contain at least one of the eleven amplicons with some amplicons crossing haplotype block boundaries. We then overlapped the target haplotype blocks with the SNPs from (16) due to the greater resolution in that study. This approach identified 204 putative SNPs in the 13 haplotype block regions.

The initial combined marker panel totaled 381 SNPS, 204 from (9) and 177 from (16). We further reduced the number of markers to those that were most contributive to separating RHB from the other populations. To achieve this, we used the minor allele frequency matrix for the combined set of 381 SNPs in a Principal Component Analysis (PCA). Using the absolute value of the loadings for the first two principal components which account for 26% of the variance in the analysis, a subset of markers with the highest contribution (>= 70% among all markers) in separation was selected. This set of outliers constituted the final testing panel of 164 markers.

### Sample processing

Performance of our test panel was validated with archived, frozen DNA of 223 samples that included the RHB and other commercial and research honey bee populations used as baseline in (9). In addition, we processed 154 novel samples collected from the 2019 RHB population as part of a yearly genotyping assay conducted by the Honey Bee Breeding, Physiology, and Genetics Research Laboratory (HBBPGRL) and 72 samples collected from three Italian honey bee (IHB) commercial and research populations for a total sample size of 449. These 2019 RHB samples (n = 154) and known IHB samples (n = 72) were considered together to examine the diversity of current populations in relation to the (9) baseline sample sets.

The method for DNA extraction for novel samples followed those described in (9) to assure comparable processing. Briefly, samples were first homogenized in lysis buffer (100 mM Tris pH 8.0, 10 mM EDTA pH 8.0, 1% SDS) and 100 mg 5-mm stainless steel beads for 3 min at a rate of 30 beats per second in a TissueLyzer II (Qiagen, Inc., Frederick, MD) and then treated with Proteinase K (20 mg/ml) at 70°C for 10 min. Protein precipitation was then completed, followed by ethanol precipitation and lyophilization. Pure genomic DNA was rehydrated in Millipore filtered and deionized dH2O and stored at −20°C until further processing.

### Assay deployment

A Fluidigm 96.96 Dynamic Array™ IFC (Fluidigm Corp, South San Francisco, CA, USA) genotyping assay was developed for all 164 target SNPs using genomic coordinates and the honey bee reference genome (21). Resulting assay chemistry, microfluidic chip, and DNA extracts were provided directly to the Functional Genomics Unit of the Roy J. Carver Biotechnology Center of the University of Illinois at Urbana-Champaign where processing was conducted according to the manufacturer’s protocol using the Fluidigm Biomark System. Briefly, a pre-amplification step was conducted on each DNA sample using a pool of the Specific Target Amplification (STA) primers and Locus-Specific Primer (LSP). This step yields a pool of amplicons that includes each of the 164 SNPs for each of the 449 samples processed. Pools where then processed on the Fluidigm 96.96 Dynamic Array™ IFC microfluidic chip in the Biomark System using the LSP and Allele-Specific Primers (ASP), which are pairs of primer sets labeled with different fluorophores.

Genotypes were called using the Fluidigm SNP Genotyping Analysis Software (version 4.5.1). We initially applied manual validation of the automatically generated fluorescence scatter plots. Using these as a guide we removed from the assay any SNP that had a poor call rate (<= 80%) or whose no template control (NTC) fluoresced in overlap with any of the genotype clusters. Lastly, any NTC with significant fluorescence (> 0.2) was removed regardless of overlap. Once these considerations were applied, we further filtered the resulting genotype calls by sample and degree of information. Specifically, we removed those samples with significant degrees of missingness (> 20%) and also those SNPs where genotypes were present, but were uninformative due to homogeneous calls, i.e. all one genotype across the entire sample set. We also used the relationship between SNP genotypes to filter out those markers with near zero variance and to prune sets of highly correlated markers to single representatives. This conservative filtering approach resulted in a final data set of 81 SNP markers and 394 samples.

### Feature extraction

**Figure 1:**
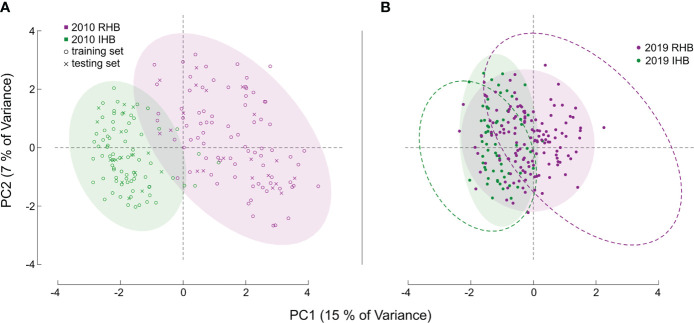
Principal Component Analysis of Sample Sets. Figures **(A, B)** both illustrate the PCA values derived from the genotype matrix of all the samples. Each point corresponds to an individual honey bee worker with distances generally corresponding to degree of genetic similarity in allele structure between individuals across all markers in our assay. For **(A)** the baseline population is shown, with color denoting known population membership of RHB (magenta), and a panel set of commercial and research honey bee population (green). The symbols for each point denote whether a specific sample was used as part of the training set (open circle) or part of the independent testing set (x symbol). For both figures, color-filled ellipses encircling 95% of samples are provided to clarify genotypic boundaries between populations. For **(B)** the novel set of samples are illustrated, like **(A)**, point color corresponds to population source, but in this set for the 2019 samples. The dashed ellipse in **(B)** is the outline of those illustrated in **(A)** and are provided as reference to illustrate the shift in genetic diversity for the 2019 samples.

A machine learning approach was used in the analysis of our final SNP panel set. The approach capitalized on having the known membership for the original mapping population which together with the genotypes of the new markers were applied to build a predictive model using a panel of classification algorithms. After filtering, our total sample size of n = 394 included n = 191 samples from the the original mapping population (9). We used these to arrive at a predictive model by 1) isolating 20% of the samples (n RHB = 20, n other = 18) as our test set and 2) using the remaining 80% (n RHB = 80, n other = 73) as our training set (**Figure 1A**). Initial performance of 7 classification algorithms including 4 random forest (23), 1 naïve bayes (24), 1 XGBoost (25) and the k-nearest neighbor was examined to identify the one with the highest predictive power for our data set.

### Model selection, tuning, and training

Data was pre-processed prior to tuning by mean-imputing missing values. In this way our genotype matrix retained dosage values that would lie within the standard 0, 1, 2 scale. Model selection and tuning was conducted concomitantly. Using the training set, we examined performance of all 7 classification algorithms each with an algorithm-specific set of parameters. Leave one out cross validation was implemented to assess the optimal model resulting from each algorithm and parameters set. These resulting models, one from each algorithm, were then compared to arrive at a final discriminant model. Of all algorithms and parameter combinations examined, the model derived from the *ranger* algorithm (26) provided the highest predictive accuracy. The *ranger* algorithm in particular was tested using a grid of specific parameters as follows: *mtry* = c(2:10), *splitrule* = c(“gini”, “extratrees”), *min.node.size* = c(2:10), where *mtry* the number variables to split at each node, *splitrule* is the splitting rule at each node, and *min.node.size* is the minimal node size. Combined, this parameter grid totaled 162 possible combinations considered within this algorithm from which we established the specific parameter combination resulting in our final classification model. Model tuning and training tests used the F-score as principal estimator of the balance between model precision and model recall, but other estimates (e.g. Accuracy, Specificity, Kappa) were also taken into consideration in our final selection. In all tests predictive power was derived using the testing set containing the 20% of the baseline population samples unused in the training. All analyses for model testing and tuning were conducted using the *caret* package in R (27). Estimates of memberships for the testing set was derived using the *predict*() function in R and these were compared using the *confusionMatrix*() function in the *caret* package.

### Performance contrast and genotype identification

Performance of our approach was contrasted with the original GSI assay by using the baseline testing set. We conducted two independent population membership predictions. One approach used our model to predict membership on the testing set (n = 38). For another approach we used the method in Burgeois et al., 2010 but excluded our testing set and then used that model to predict membership for those 38 samples. In this way, the testing set represents samples with known population membership, membership predicted by our model, and also membership predicted by the classification approach in (9). In this way we compared performance by contrasting each model’s prediction to the known membership to arrive at model-specific accuracy estimates. We also statistically examined how different these predictions were from the know differences through a chi-square test of independence.

**Figure 2:**
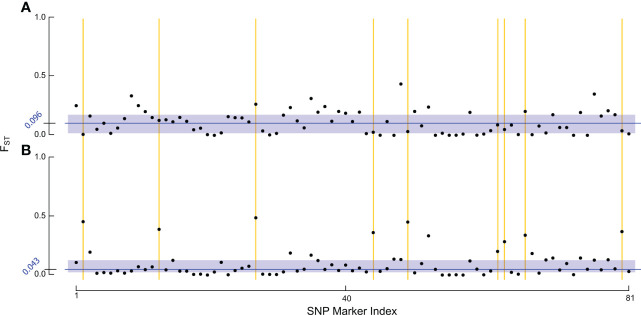
Analysis of F_ST_ estimates. The figure set illustrates the F_ST_ estimates for each of: **(A)** baseline sample set, and **(B)** novel sample set using population source to identify samples in both sets. Each point in the figure represents the F_ST_ estimate for one of our final 81 SNP markers within the respective RHB v. IHB population contrast. The blue rectangles encapsulate the interquartile range of F_ST_ values. The median F_ST_ value for each population is indicated by the dark blue line and exact value is given in the axis also in blue. The yellow lines highlight some of the markers in our panel that have become more divergent in the 2019 population.

We also used an additional novel sample set (n = 203 post filtering) containing recently collected representatives from the RHB and other commercial honey bee populations to examine how allelic preferences may have shifted from sampling in (9) and now (**Figure 1B**). Specifically, the markers in our panel represent genomic regions with the greatest genetic divergence between the RHB and other honey bee populations. Any changes in allelic profile of these markers would then suggest possible population-wide events such as introgression. Furthermore, this set of samples has population membership that is known by collection source, but not by genetic identity, providing a unique opportunity to examine divergences of the predictive membership to the collection source. For specific analysis of shifts in genetic divergence we used a contrast of population- and SNP-based F_ST_ values in the baseline set against those of the novel sample set (**Figure 2**). Estimates for F_ST_ (28) were derived using the *snpgdsFst()* function part of the SNPRelate package in R (29).

## Results

### Model selection, tuning, and training

Analysis of our seven model panel using leave one out cross validation showed the *ranger* (26) algorithm was the optimal option (best tuned model cross validated F score = 0.9938, Accuracy = 0.9935, and Kappa = 0.9869). Model tuning determined optimal parameters were: mtry = *1*, splitrule = *extratrees*, and min.node.size = *3*. We examined model performance by predicting population membership of the testing set (n = 38) which had not been used for model training and contrasted predictions with their known population membership. For this subset of data our model had an Accuracy and F score of 1.0, accurately predicting the membership for the entire set.

### Performance contrast and genotype identification

Contrast in the performance of the original GSI assay and the expanded GSI show that both arrive at predicted membership values that do not significantly differ from known membership in the testing set (original GSI X^2^ = 3.744, df = 1, p-value = 0.053; expanded GSI X^2^ = 34.095, df = 1, p-value = 5.25 E^-9^). Comparison of performance metrics do show distinct differences between approaches. The expanded GSI correctly predicted values to a high degree (both Accuracy and F score at 1.0). Predictive performance of the original GSI had lower Accuracy (0.6842) and F score (0.7000) values, misclassifying both RHB samples (n = 6) and IHB samples (n = 6).

**Figure 3:**
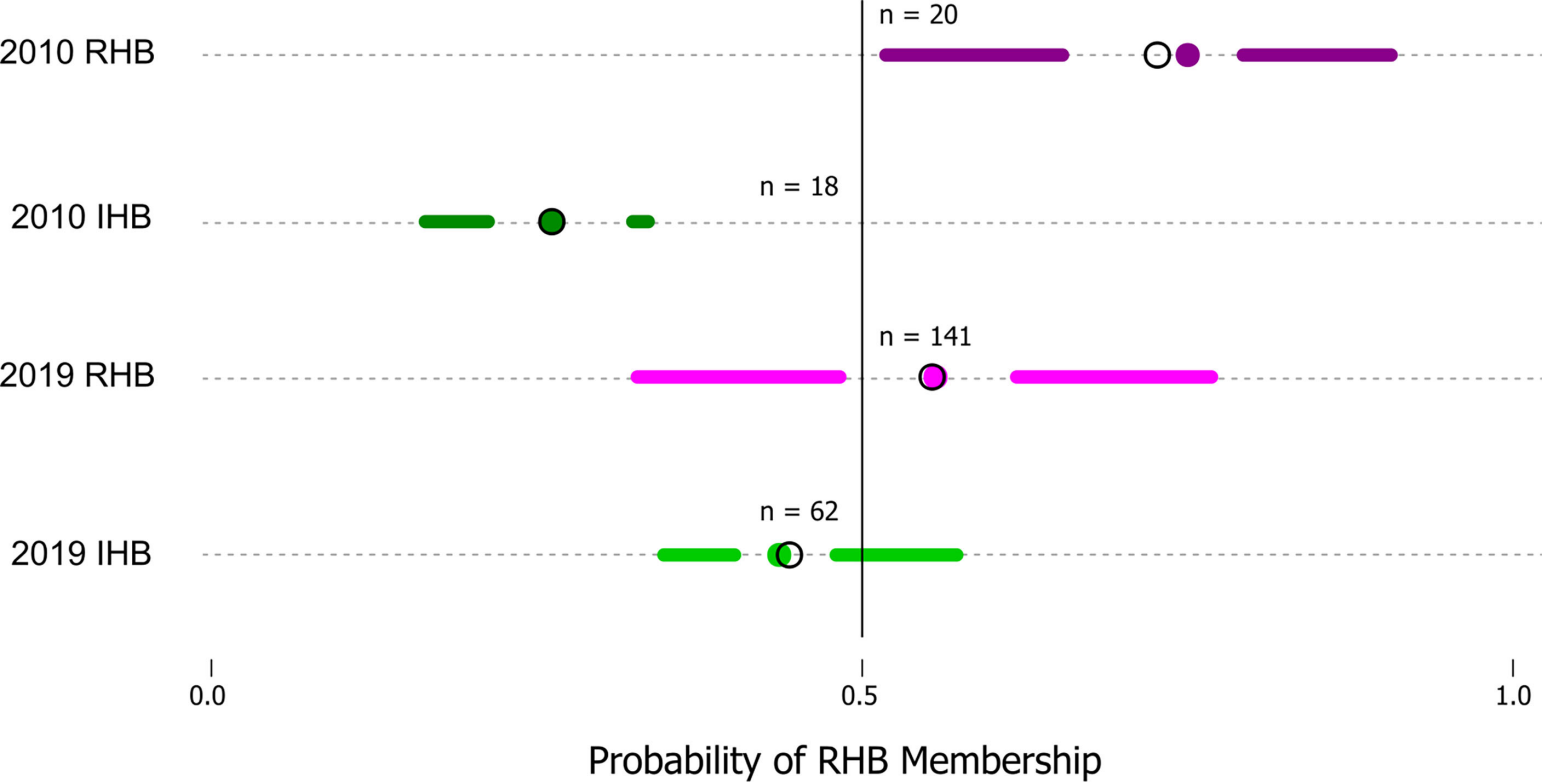
Distribution of probabilities of RHB membership classification. The figure is a simplified boxplot of the distribution of probabilities that a sample would be classified as part of RHB (x-axis) as estimated by our approach. The y-axis highlights our data groups with two reference sample sets (2010 RHB and IHB) are provided which constitute the predicted probability values for the testing set (n = 38) and two additional sets (2019 RHB and IHB) which represent our novel samples from the 2019 populations. A solid line denotes the threshold value (0.5) above which a sample would be classified in as part of one group or another. For all plots the segments span from the minima to the maxima probability value in each data set while the gap highlights the interquartile range. The colored points identify the mean and the open circle the median in each of the groups.

Model performance was also examined in the 2019 novel sample set (n = 203) containing representatives from the current RHB population (n = 141) as well as from other commercial honey bee populations (n = 62). These samples represent a set with known collection source, but unknown genetic identity. We contrasted membership as defined by collection source against the membership predicted by our model to arrive at model performance estimates. Model parameters showed a lower Accuracy (0.7143) and F score (0.7642). Further analysis also showed the greatest difference in model performance was between Sensitivity (true positive rate) value (0.8952) and Specificity (true negative rate) value (0.5204). Largely, the difference between these metrics stemmed from the model misclassifying a larger proportion of RHB samples (0.67) compared to the IHB samples (0.18). The distribution of classification probabilities (**Figure 3**) provided a greater resolution to the differences between the collection sources, showing a pattern concordant with the shift in genetic variation previously observed in the PCA between the 2010 and 2019 samples (**Figure 1B**). These population-specific patterns are likely not an artifact, but may reflect actual changes in genetic diversity in the RHB population from 2010 to 2019 and agree with previous studies on introgression events in this population (13).

To test whether the lower true positive rate in RHB reflected shifts in the allelic profile of the population, we examined genetic divergence between samples across our target markers (**Figure 2**). For the F_ST_ estimation we contrasted RHB and IHB samples using the population source as our membership classifier. Estimates for the baseline set were derived using all the samples, both test (n = 38) and training (n = 153), in this way the combined set serves as a reference of the genetic variation across both RHB and IHB populations in 2010 (**Figure 2A**). Similarly, all samples were used in the novel set (**Figure 2B**). Both population (average F_ST_ across the markers) and SNP-specific F_ST_ values were derived and examined. Results show that the population-level estimates were comparable for baseline (F_ST_ = 0.1154) and novel set (F_ST_ = 0.1109). The pattern was more distinct in the SNP-specific F_ST_ values across the sample sets. Overall, there was a broad reduction in F_ST_ values of the novel set (**Figure 2B**) with baseline set having a median F_ST_ value of 0.096, and the novel set median at 0.043. The similarity in population F_ST_ estimates is explained in that divergence has actually increased in a small set of markers in our panel (**Figure 2**). These results frame that genetic diversity has changed unevenly across the markers that constitute our panel assay with most markers in having a lower degree of allelic divergence in the novel set while a smaller portion of the markers have become more divergent (**Figure 2**).

## Discussion

Here we present an expanded GSI assay to facilitate discerning population membership in the RHB breeding population. Our approach improves the resolution of the original GSI assay (9) by increasing the number of segregating markers considered nearly 10 fold. The final model using the *ranger* algorithm (26) also shows a significant gain in predictive accuracy with values of 1.0 using our approach in contrast to the original GSI accuracy values of 0.67 on the same testing set of samples. Overall, the discriminant value of this assay is robust and when applied to samples within a colony can reliably identify those most similar to the referenced 2010 RHB population.

Performance in our independent testing set was high, and likely driven by the degree of genetic differentiation between samples from the 2010 population (**Figures 1A**, **3**) which has been reduced in the 2019 populations (**Figures 1B**, **3**). This distinction was initially observed in the PCA of genotype distribution within our markers set (**Figure 1**), where the 2019 RHB population seems to have moved closer to the allelic profile of the IHB samples (**Figures 1B**, **3**). This shift, within the framing of this subset of markers, coincides with and independently confirms the introgression event previously reported (13). Marker-specific analysis concurred with our PCA results (**Figure 2**). Indeed, when we look at estimates of divergence (F_ST_) for each marker in both data sets, we see a consistent lower median F_ST_ across the markers in the novel 2019 set (**Figure 2**).

The genetic shift described is directly connected to the differences in predictive performance where a large proportion (0.67) of samples collected from the 2019 RHB population are classified as IHB by our model. This is not an artifact, but rather the distribution of classification probabilities provided by our model reflect that indeed there is a greater range of 2019 RHB in the present population with lower than 0.5 likelihood membership in the RHB cluster (**Figure 2**). However, though there has been a shift in genetic variation, there remains a proportion of the RHB population that still resembles the 2010 population. If restoring the population’s genetic identity is a desired goal by RHB breeders, the higher resolution and throughput afforded by our expanded GSI over the original GSI could further facilitate the effort.

The method outlined provides a high throughput approach to the accurate discrimination of the RHB population from the broader genetic diversity in commercial honey bees in the U.S. Future work will aim to further improve the assay by expanding the reference sample set to include honey bee populations beyond those in commercial use, towards a more robust identification of the RHB breeding population. Ultimately, we expect this tool to be imminently useful to RHB breeders in their efforts. The approach as outlined also has practical potential beyond similar applications in other distinct honey bee populations. Indeed, this method can be directly applied to trait-based selection by a simple exchange of the markers used in the panel once target markers with robust genetic correlations to traits of interest are discovered.

## Data availability statement

The datasets presented in this study can be found in online repositories. The names of the repository/repositories and accession number(s) can be found below: https://datadryad.org/stash, https://doi.org/10.5061/dryad.zcrjdfngf.

## Author contributions

AA and LB conceptualized the project, compiled reference datasets, and conducted analyses class probability analyses. AA established experimental design, developed machine learning approach and conducted model testing. Both authors contributed to the article and approved the submitted version.

## Funding

This study was supported by the USDA-ARS 2020 Innovation Fund award.

## Acknowledgments

We like to acknowledge Dr. Mark Band and the Functional Genomics Unit of the Roy J. Carver Biotechnology Center of the University of Illinois at Urbana-Champaign for their aid and support in this study

## Conflict of interest

The authors declare that the research was conducted in the absence of any commercial or financial relationships that could be construed as a potential conflict of interest.

## Publisher’s note

All claims expressed in this article are solely those of the authors and do not necessarily represent those of their affiliated organizations, or those of the publisher, the editors and the reviewers. Any product that may be evaluated in this article, or claim that may be made by its manufacturer, is not guaranteed or endorsed by the publisher.
